# Pathophysiology of Obstructive Sleep Apnea in Aging Women

**DOI:** 10.1007/s40675-021-00218-x

**Published:** 2021-10-03

**Authors:** Qingchao Qiu, Jason H. Mateika

**Affiliations:** 1 John D. Dingell Veterans Affairs Medical Center, 4646 John R (11R), Room 4333, Detroit, MI 48201, USA; 2 Department of Physiology, Wayne State University School of Medicine, Detroit, MI 48201, USA; 3 Department of Internal Medicine, Wayne State University School of Medicine, Detroit, MI 48201, USA

**Keywords:** Loop gain, Arousal threshold, Obstructive sleep apnea, Post-menopause

## Abstract

The following review is designed to explore the pathophysiology of sleep apnea in aging women. The review initially introduces four endotypes (i.e., a more collapsible airway, upper airway muscle responsiveness, arousal threshold, and loop gain) that may have a role in the initiation of obstructive sleep apnea. Thereafter, sex differences in the prevalence of sleep apnea are considered along with differences in the prevalence that exist between younger and older women. Following this discussion, we consider how each endotype might contribute to the increase in prevalence of sleep apnea in aging women. Lastly, we address how modifications in one form of respiratory plasticity, long-term facilitation, that might serve to mitigate apneic events in younger women may be modified in aging women with obstructive sleep apnea. Overall, the published literature indicates that the prevalence of sleep apnea is increased in aging women. This increase is linked primarily to a more collapsible airway and possibly to reduced responsiveness of upper airway muscle activity. In contrast, modifications in loop gain or the arousal threshold do not appear to have a role in the increased prevalence of sleep apnea in aging women. Moreover, we suggest that mitigation of long-term facilitation could contribute to the increased prevalence of sleep apnea in aging women.

## Introduction to Sleep Disordered Breathing

**Fig. 1:**
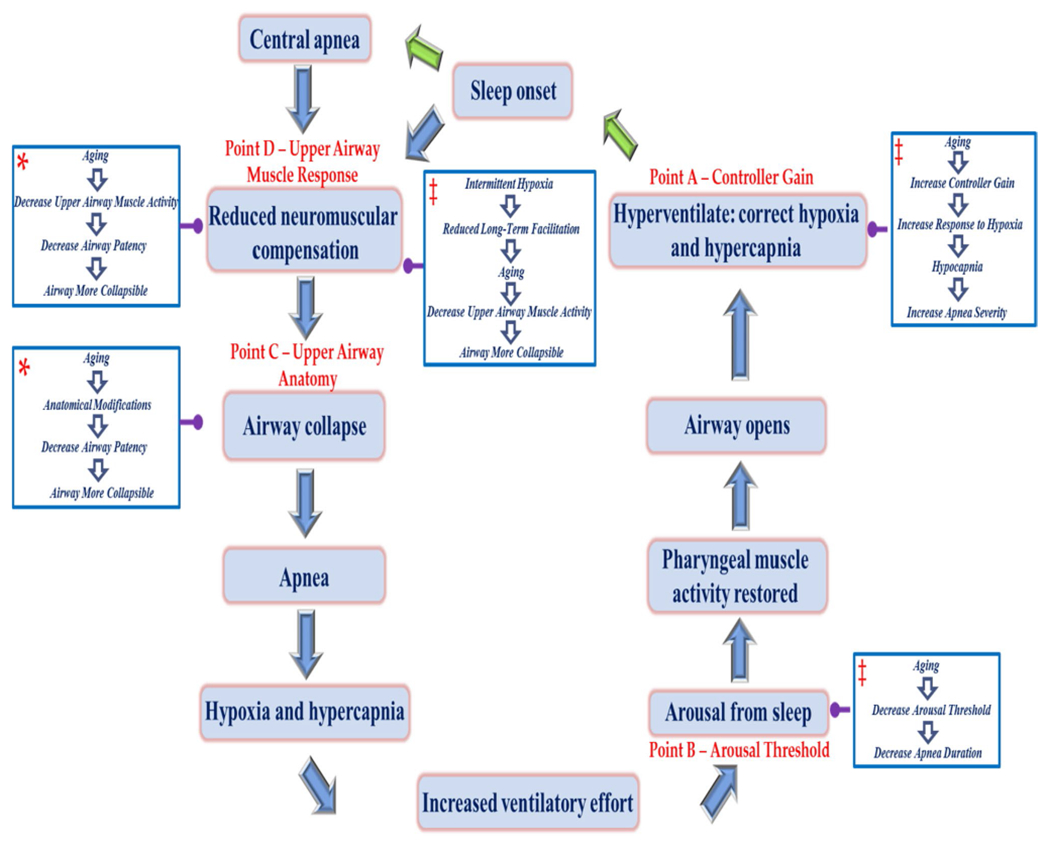
Schematic diagram showing the sequence of events leading to the development of a central and/or obstructive apnea, and subsequent events that re-establish patency of the upper airway. Various points along the pathway (see points A–D) are highlighted. At each of these points, the mechanisms that contribute to sleep apnea (i.e., controller gain, arousal threshold, upper airway anatomy/collapsibility, and upper airway muscle responsiveness) are highlighted. In addition, the potential impact that aging in women (see boxes with blue outline and white fill) might have on the various mechanisms and ultimately the prevalence/severity of sleep apnea are highlighted. For example, aging in women could lead to upper airway anatomical modifications leading to increased collapsibility of the upper airway. Please note that the asterisk within the blue box indicates that there is published data which supports a link between age in women and the pathophysiological mechanism that impacts the prevalence and severity of sleep apnea. On the other hand, double dagger indicates that there is little evidence or no published evidence to indicate that these mechanisms are responsible for the increased prevalence of apnea in aging women

Obstructive sleep apnea is a disorder associated with persistent collapse or narrowing of the upper airway during sleep. Obstructive sleep apnea has been linked to several detrimental outcomes including excessive daytime sleepiness, enhanced sympathetic nervous system activity, increased cardiovascular risk, and impaired cognition [1–3]. There are at least four principal endotypes that contribute to sleep apnea: (i) increased collapsibility of the upper airway (obstructive sleep apnea); (ii) a blunted upper airway muscle response to changes in the partial pressure of carbon dioxide (obstructive sleep apnea); (iii) a low arousal threshold; and (iv) instability of the ventilatory control system (i.e., increased loop gain) [1, 4, 5] (Fig. 1 in points A–D). These endotypes may be altered by many physiological variables including sex and age. This short review will focus primarily on the role that these endotypes have in the pathophysiology of obstructive sleep apnea in aging women.

## Sex and Age Differences in the Prevalence of Sleep Disordered Breathing

Obstructive sleep apnea is more prevalent in men compared to women [6–11], even when body mass index is controlled [12]. The disparity in the prevalence of obstructive sleep apnea tends to be more pronounced in clinic-based studies [11] compared to community-based studies [10]. The discrepancy between men and women is also dependent in part on the criteria used to define the disorder. A greater discrepancy is evident if a single criterion is used (e.g., an apnea/hypopnea index ≥ 5 episodes per hour of sleep) [10]. In contrast, the discrepancy is reduced when additional criteria to define sleep apnea are included (i.e., symptoms of day time sleepiness) [10]. Variations in the prevalence between the sexes might occur because sleep disordered breathing manifests differently in men compared to women. In contrast to men, investigators have suggested that women experience episodes of increased upper airway resistance that are not accompanied by significant airway occlusion [13–15]. These episodes do not meet the formal definition of an apnea or hypopnea, but nevertheless increase the work of breathing, cause disrupted sleep, and ultimately result in daytime cognitive dysfunction and fatigue, depression, and insomnia. These are symptoms that women with sleep disordered breathing are more likely to experience [16].

Nonetheless, when the apnea/hypopnea index is used to define sleep disordered breathing, the prevalence of sleep apnea is reduced in women compared to men during non-rapid eye movement sleep [17]. The reduction is due to a significant decrease in the number of apneic events. Consequently the apnea/apnea + hypopnea ratio is reduced in women [18]. In addition, the duration of apneic events is shorter in women compared to men [19]. The reduced duration in women may occur because they are more likely to experience cortical arousal [20]. The reduced apnea duration might also be linked to the finding that declines in oxygen saturation are diminished during apneic events in women compared to men [12]. In contrast to non-rapid eye movement sleep, disruptive sleep events are more evident in rapid eye movement sleep in women [16, 21, 22]. Thus, women tend to be more protected from sleep disordered breathing during non-rapid but not rapid eye movement sleep.

Differences in the apnea/hypopnea index that exist between men and women decrease with age. The primary reason is because the apnea/hypopnea index during both non-rapid eye and rapid eye movement sleep increases more dramatically as women age [21]. Likewise, some studies have reported that the prevalence of sleep disordered breathing during rapid eye relative to non-rapid eye movement sleep decreases with age more precipitously in women compared to men [16, 21], while other studies have indicated that this difference remains constant over the life span. This discrepancy could be due to variations in the definition of rapid eye movement-related sleep disordered breathing that was employed in the completed studies. Nevertheless, collectively the published findings indicate that sleep disordered breathing increases in aging women. The mechanistic underpinnings of the modifications in aging women are addressed in the following sections.

## Aging Women, Loop Gain, and Obstructive Sleep Apnea

The stability of the ventilatory control system plays a significant role in the pathophysiology of obstructive sleep apnea. An unstable ventilatory control system results in fluctuations in ventilatory drive, ultimately leading to an unstable upper airway and airway collapse [1, 23, 24]. Ventilatory stability is quantified using an engineering concept referred to as loop gain. Loop gain is comprised of controller gain (Fig. 1 — point A) and plant gain, and is a measure of the response of the ventilatory control system to one or more inputs (e.g., hypoxia, hypercapnia) [1, 25]. An unstable ventilatory control system is characterized by a loop gain value greater than one. Controller gain is altered by modifications in chemoreflex sensitivity [26]. The greater the ventilatory response to a given level of hypoxia or hypercapnia, the greater the controller gain. Measures of plant gain denote the efficiency of the ventilatory system in the maintenance of homeostatic carbon dioxide levels [27]. Typically, an equilibrium between the arterial partial pressure of carbon dioxide and ventilation is maintained. As the arterial partial pressure of carbon dioxide increases, an accompanying increase in ventilation is initiated by the peripheral and central chemoreceptors. Activation of the receptors returns the arterial partial pressure of carbon dioxide to a homeostatic equilibrium [27].

If the ventilatory response to increases in carbon dioxide and decreases in oxygen, induced by an apnea, is inappropriately high (NB because of an increase in chemoreflex sensitivity) upon arousal from a breathing event, the outcome will be a significant reduction in carbon dioxide (i.e., hypocapnia) below homeostatic levels [28] (Fig. 1 — point A). If the induction of hypocapnia persists on the return to sleep, a central apneic event (Fig. 1) coupled to an obstructive event will often occur (Fig. 1) and these events will present in a cyclical manner [28].

Given that an increase in loop gain may lead to increases in apnea severity, it is possible that this mechanism is responsible for increases in apneic events in aging women (Fig. 1 — point A). More specifically, one would anticipate that loop gain, or controller gain (i.e., chemoreflex sensitivity) which is a component of loop gain, would gradually increase in aging women. Indeed, Chowdhuri and collegues reported that the hypoxic ventilatory response, which is a surrogate measure of chemoreflex sensitivity, is greater in older males and females compared to younger adults [29]. Hormonal modifications associated with menopause (i.e., reductions in estrogen and/or progesterone) might be the underlying mechanism responsible for the increase in loop gain and consequently apnea severity. In support of this possibility, some studies have reported that lower levels of estradiol and/or progesterone are coupled to increased severity of sleep disordered breathing [30]. Moreover, sleep apnea was reported to be more prevalent in post-menopausal women that do not receive hormone replacement therapy compared to pre- or post-menopausal women that receive hormone replacement therapy [7].

On the other hand, no correlation between hormone levels and the hypoxic ventilatory response is evident in women [29]. Moreover, measures obtained from adults over 60 years of age, including older women, showed that loop gain was blunted compared to younger adults (i.e., < 40 years of age) [31]. This latter finding indirectly supports studies which indicated that female sex hormones enhance the ventilatory response to hypoxia or hypercapnia [32–34]. In addition, exogenous progesterone in combination with estrogen increases the hypoxic and hypercapnic ventilatory responses in post-menopausal women [33, 35]. Similarly, progesterone alone increases the ventilatory response to hypoxia and hypercapnia in males [34]. If increases in estrogen and progesterone increase the hypoxic and hypercapnic ventilatory response, the severity of sleep apnea would be expected to be lower in post-menopausal women, which is not the case. Lastly, a recent meta-analysis of the published literature indicated that changes in female sex hormones during menopause do not explain the increase in sleep disordered breathing in midlife women and that conclusions on the effect of hormone replacement therapy on sleep disordered breathing cannot be drawn from the current literature [36]. Overall the published data indicates that modifications in loop gain or chemoreflex sensitivity are likely not the principle mechanism behind increases in apnea severity that are evident in aging women.

## Aging Women, the Arousal Threshold and Obstructive Sleep Apnea

Arousal from sleep is often associated with the termination of breathing events in patients with obstructive sleep apnea. Arousals play an essential role in the restoration of upper airway patency because they are accompanied by increased upper airway dilator muscle activity [37]. Despite this advantageous response, arousal from sleep caused by minor increases in ventilatory drive may occur in patients with a low arousal threshold. A low arousal threshold (Fig. 1 — point B) coupled to a high loop gain (Fig. 1 — point A), or ventilatory response to arousal, could lead to destabilized breathing and result in the perpetuation of apneic events [38], as described in the previous section. The increase in sleep apnea severity in aging women suggests that reductions in the arousal threshold from pre- to post-menopause might contribute to the increase (Fig. 1 — point B). However, as outlined below, there is limited evidence to suggest that there is a causal link between reductions in the arousal threshold and increases in apnea severity during the transition from pre- to post-menopause.

Respiratory events are significantly shorter in women compared to men (see “Sex and Age Differences in the Prevalence of Sleep Disordered Breathing” section)[19]. The shorter duration could be a consequence of a reduced arousal threshold [39]. Although there is evidence to support this contention, this sex difference may vary depending on the age of the population. Women that are of pre-menopausal age appear to be more susceptible to cortical arousal [20]. In contrast, women of post-menopausal age are less susceptible to arousal compared to pre-menopausal women. This suggestion is supported by findings which showed that post-menopausal women tend to sleep longer and have deeper sleep than pre- and peri-menopausal women [40]. Likewise, post-menopausal women have a lower arousal burden compared to elderly men [41]. However, it bears mentioning that in elderly women, the susceptibility to cardiovascular mortality is greater compared to men despite the lower arousal burden [41].

The findings of a lower arousal burden in aging women are also coupled with an increase in event duration [19]. The increase in event duration could be a consequence of modifications in the arousal threshold. However, Edwards and colleagues reported no modifications in the arousal threshold with age [31]. Instead, it was suggested that an increase in circulatory delay could be responsible for increases in event duration [19]. Thus, reductions in the arousal threshold are not likely responsible for differences in apnea severity between pre- and post-menopausal women. Indeed, the preponderance of evidence seems to indicate that the arousal threshold either remains unchanged or increases from pre- to post-menopause. Consequently, increases in apnea severity and duration are not likely due to variations in the arousal threshold in aging women.

## Sex Differences in Upper Airway Collapsibility and Muscle Responsiveness

The human upper airway is predominantly composed of muscle and soft tissues and is largely devoid of rigid, bony structures. As a result, the upper airway is susceptible to collapse from various anatomical or physiological perturbations. Individuals with a narrow upper airway, and/or increased tissue pressure around the upper airway due to fat deposits, have a greater likelihood of airway collapse compared to an airway with a larger diameter [42]. The pressure at which the upper airway collapses is referred to as the critical closing pressure. A more positive critical closing pressure is associated with increased pharyngeal collapsibility [43].

The increased prevalence in apnea severity in men versus women may in part be due to alterations in airway caliber including the size and stiffness of the pharyngeal lumen, and the pressure gradient across the pharyngeal wall. Malhotra et al. [44] and other investigators [45, 46] have shown that the pharyngeal airway in healthy men is longer compared to women independent of height. A longer airway tends to be more susceptible to collapse [44, 47]. Likewise, thickening of the soft tissue located on the lateral walls of the airway may increase the susceptibility of airway collapse by increasing extraluminal and reducing intraluminal pressure [48]. Whittle et al. [49] reported that the volume of soft tissue was greater in men compared to women, which could lead to an increase in the mechanical load on their upper airway. The majority of this volume was not adipose tissue. Dancey et al. [50] also reported that differences in neck circumference could contribute to variations in the caliber of the upper airway in men compared to women. The increase in neck circumference in men could be due in part to an increase in fluid shift from the legs to the thorax and neck in the supine position that occurs as a consequence of increases in intrathoracic pressure that is associated with sleep apnea [51]. Likewise, differences in pharyngeal cross-sectional area may be anticipated in men and women, since patients with obstructive sleep apnea have a significantly smaller pharyngeal cross-sectional area compared to healthy individuals [49, 52]. However, the majority of the literature indicates that cross-sectional area is similar in men and women either before [49, 52, 53] or after correcting for body surface area [54, 55].

Given that mechanical properties differ between men and women, one might anticipate that several outcome measures that reflect upper airway mechanics (i.e., upper airway collapsibility, resistance, and compliance) vary in men compared to women with sleep apnea. Indeed, upper airway collapsibility was reported to be greater in men compared to women after controlling for body mass index, age, and apnea severity [56]. Likewise, Pillar et al. [47] reported that healthy men exhibited a significantly higher increase in pharyngeal resistance in response to inspiratory loading during non-rapid eye movement sleep compared to women, even though the ventilatory response to the load was similar. As is the case with the critical closing pressure, the increase in upper airway resistance found in men may be indicative of a more collapsible airway.

The reduced resistance in women might be the result of increased tonicity of upper airway muscles (i.e., genioglossus muscle). Indeed, upper airway muscle activity has been shown to be positively correlated with progesterone levels in female subjects [57]. This mechanistic difference between the sexes could account for the increased prevalence of apneic events in non-rapid eye movement sleep in men compared to women. Conversely, muscle atonia during rapid eye movement sleep could negate the effects of sex hormones on airway patency leaving women more susceptible to airway collapse, increased resistance, and disordered breathing.

In contrast to the reported sex differences in airway collapsing pressure and airway resistance outlined in the previous two paragraphs, Pillar et al. [47] and Rowley et al. [58] reported that the critical closing pressure and pharyngeal resistance was similar in men and women. This suggestion was supported by additional findings of Rowley et al. [55], which showed that retropalatal compliance was similar in men and women during wakefulness and non-rapid eye movement sleep when neck circumference was controlled.

Collectively, increased airway length and neck circumference have been shown to be consistently greater in men compared to women. These differences could be responsible in part for the increased propensity for upper airway closure in men compared to women. However, because outcome measures of collapsibility and resistance are not entirely consistent, it is possible that modifications in neurochemical control of upper airway muscle activity contribute to sex differences in the prevalence of sleep-disordered breathing.

## Aging Women, Upper Airway Collapsibility, and Muscle Responsiveness

The modifications in mechanical properties that may in part be responsible for sex differences in apnea severity suggest that similar modifications could be evident in aging women (Fig. 1 — point C). Indeed, post-menopausal women tend to have a higher body mass index, neck circumference, and waist-to-hip ratio, along with higher fat deposition in the upper body and trunk area compared to pre-menopausal women [59]. The modifications in visceral adiposity could provoke obstructive sleep apnea via excessive fat disposition in the parapharyngeal space accompanied by an increased work of breathing [60]. Likewise, increases in retropalatal and retroglossal airway length have been reported in the elderly [61]. The lengthening of the airway could be due to a progressive descent of the larynx and epiglottis with aging. Published work has reported that the length of the pharyngeal airway changes on average 4.2 mm between younger and older men and 8.0 mm between younger and older women [60]. When stratified by gender, a significant correlation is seen in women, but not in men [60]. These changes in women could contribute in part to the increases in apnea severity observed during the transition from pre- to post-menopause. Thus, modifications associated with aging and obesity, which impact both sexes in some cases, and women specifically in other cases, might lead to greater collapsibility of the airway in aging women.

Studies have shown that age independently influences both upper airway collapsibility [31, 56] and the increase in pharyngeal resistance during sleep [62], whereas body mass index and gender explain minimal variance at either end point [63]. Indeed, drug-induced sleep endoscopy revealed that age predisposes older individuals to multisite and multilevel collapse of the upper airway [63]. This predisposition may be independent of sex [63]. However, using endoscopy, Koo and colleagues reported that the severity of airway obstruction is greater in post-menopausal women than in pre-menopausal women, particularly at retropalatal and retro-lingual sites [64]. In addition, determination of the pressure required to induce collapse of the upper airway either by the administration of pressure [56], or the use of computer modelling [31], has revealed that the collapsibility of the pharyngeal airway worsens with aging among patients with obstructive sleep apnea, a finding that was independent of sex. The worsening of sleep apnea due to modifications in upper airway anatomy and collapsibility in older adults, and particularly post-menopausal women, may be a unique phenotype specific to this age group, since modifications in loop gain and arousal threshold do not appear to explain differences in apnea severity between pre- and post-menopausal women [31].

Differences in apnea severity between pre- and post-menopausal women could also be caused by modifications in upper airway muscle responsiveness (Fig. 1 — point D). Indeed, sex hormones might affect the function of the pharyngeal dilator muscles, influencing airway collapsibility, and respiratory control [57]. This suggestion is supported by findings which showed that the pattern of airway obstruction and upper airway muscle activity are different between pre-menopause and post-menopause [57, 64]. In awake women, the activity of the genioglossus muscle, which is a pharyngeal dilating muscles, was lower after compared to before menopause [57]. The decline in progesterone that accompanies post-menopause contributed to the decreased genioglossus activity [57]. Moreover, in a subgroup of 8 women, genioglossus activity increased significantly after 2 weeks of hormone replacement therapy [57], which supports the possibility that a reduction in reproductive hormones could be linked to an increased risk of obstructive sleep apnea in post-menopausal women [57]. Likewise, by means of electromyography, studies found that age-related weakening in the genioglossus muscle increased the susceptibility to tongue collapse in humans [65].

Thus, there is some evidence to suggest that aging and the transition to post-menopause attenuate the response of the genioglossus muscle to negative pressure and hypoxia [60, 66]. However, it should be noted that these studies were conducted in healthy participants during wakefulness [60, 66]. Consequently, it remains to be determined if these deficits are evident during sleep in patients with obstructive sleep apnea and whether reduced muscle responses that occur with aging and/or the transition to post-menopause translate into airflow impairments. To date, Patil and collegues found no age-related change in the dynamic responses to upper airway obstruction during sleep in patients with obstructive sleep apnea [67]. Likewise, Edwards and colleagues using a computer-based modelling system indicated that upper airway gain, which is a non-invasive marker of upper airway muscle sensitivity, was not altered with age and was independent of sex [31].

## Aging Women, Respiratory Plasticity, and Obstructive Sleep Apnea

Exposure to intermittent hypoxia, which is a hallmark of sleep apnea, initiates a form of respiratory plasticity referred to as long-term facilitation [68–72]. Long-term facilitation is characterized by a sustained increase in respiratory motor output (i.e., hypoglossal or phrenic nerve output) or ventilation that persists during normoxia following exposure to intermittent hypoxia [69]. Long-term facilitation has been documented in several animal models [69], and in healthy humans [73–76] and humans with sleep apnea [73, 74, 77, 78] or spinal cord injury [79]. Long-term facilitation has been documented during wakefulness [73–77] and sleep [74] in humans.

Long-term facilitation of hypoglossal or upper airway muscle activity might mitigate sleep apnea [68, 70–72], particularly if loop gain or chemoreflex sensitivity is blunted. Specifically, sustained increases in upper airway muscle activity initiated by exposure to intermittent hypoxia could lead to enhanced patency of the upper airway and mitigate apneic events (Fig. 1 — point D). Indeed, studies have shown that exposure to intermittent hypoxia leads to sustained increases in genioglossus muscle activity in humans during both wakefulness [80] and sleep [81]. Moreover, exposure to intermittent hypoxia during sleep results in reductions in airway resistance and upper airway collapsibility in humans [82]. These latter findings are assumed to be a consequence of long-term facilitation of upper airway muscle activity.

Given the increased prevalence of sleep apnea in post-menopausal females, the magnitude of long-term facilitation may be greater in young and middle aged pre-menopausal females compared to post-menopausal females (Fig. 1 — point D — blue box). This hypothesis is supported indirectly by studies completed in middle aged female rats. The magnitude of long-term facilitation varied within the estrous cycle, reaching a peak during diestrus [83], a stage characterized by high progesterone levels [84]. The correlation between the magnitude of long-term facilitation and the estrus cycle may be related to serotonin levels since serotonin levels modulate throughout the rat estrus cycle reaching a peak during diestrus [85]. This possibility is important since serotonin is one of many neuromodulators released in response to intermittent hypoxia to initiate long-term facilitation [86].

No studies to our knowledge have directly explored the relationship between pre- and post-menopause and the magnitude of long-term facilitation of upper airway muscle activity in women. However, there are some indirect findings which suggest that long-term facilitation of upper airway muscle activity may be reduced in aging women. Chowdhuri and colleagues reported that upper airway resistance following exposure to intermittent hypoxia was similar to baseline in a group of elderly men and women [29]. This finding suggests that long-term facilitation of upper airway muscle activity was not initiated in women of post-menopausal age. In contrast, exposure to intermittent hypoxia in women of pre-menopausal age consistently leads to decreases in upper airway resistance [87]. Thus, some evidence suggests that reductions in sex hormones with post-menopause might lead to mitigation or elimination of a form of plasticity that could contribute to mitigating sleep apnea. However, additional work is required to explore if reductions in the magnitude of long-term facilitation contributes in part to the increase in apneic events in aging women.

## Summary

In summary, increases in apnea severity are evident in aging women. This increase may be mediated by a number of possible mechanisms which include modifications in anatomy, reductions in upper airway muscle responsiveness, reductions in the arousal threshold, and increases in loop gain. Moreover, compensatory mechanisms that might serve to mitigate sleep apnea might also be modified with aging. Presently, the published literature indicates that increases in apnea severity in aging women may be caused primarily by modifications in upper airway mechanical properties. On the other hand, there is little supporting evidence to suggest that modifications in loop gain, the arousal threshold, or upper airway muscle responsiveness are principally responsible for the modifications in apnea severity in aging women.
